# Improving Quality of Carotid Interventions: Identifying Hospital-Level Structural Factors that can Improve Outcomes

**DOI:** 10.1016/j.avsg.2020.09.066

**Published:** 2021-04

**Authors:** Kamran Gaba, Dylan Morris, Alison Halliday, Richard Bulbulia, Prem Chana

**Affiliations:** 1 Nuffield Department of Population Health, Medical Research Council Population Health Research Unit, University of Oxford, Oxford, UK; 2 Nuffield Department of Population Health, Clinical Trial Service Unit and Epidemiological Studies Unit, University of Oxford, Oxford, UK; 3 Nuffield Department of Surgical Sciences, University of Oxford, Oxford, UK; 4 Department of Academic Surgery, St Mary's Hospital, Imperial College, London, UK

## Abstract

**Background:**

“Structural factors” relating to organization of hospitals may affect procedural outcomes. This study's aim was to clarify associations between structural factors and outcomes after carotid endarterectomy (CEA) and carotid endarterectomy stenting (CAS).

**Methods:**

A systematic review of studies published in English since 2005 was conducted. Structural factors assessed were as follows: population size served by the vascular department; number of hospital beds; availability of dedicated vascular beds; established clinical pathways; surgical intensive care unit (SICU) size; and specialty of surgeon/interventionalist. Primary outcomes were as follows: mortality; stroke; cardiac complications; length of hospital stay (LOS); and cost.

**Results:**

There were 11 studies (*n* = 95,100 patients) included in this systematic review.

For CEA, reduced mortality (*P* < 0.0001) and stroke rates (*P* = 0.001) were associated with vascular departments serving >75,000 people. Larger hospitals were associated with lower mortality, stroke rate, and cardiac events, compared with smaller hospitals (less than 130 beds).

Provision of vascular beds after CEA was associated with lower mortality (*P* = 0.0008) and fewer cardiac events (*P* = 0.03). Adherence to established clinical pathways was associated with reduced stroke and cardiac event rates while reducing CEA costs.

Large SICUs (≥7 beds) and dedicated intensivists were associated with decreased mortality after CEA while a large SICU was associated with reduced stroke rate (*P* = 0.001).

Vascular surgeons performing CEA were associated with lower stroke rates and shorter LOS (*P* = 0.0001) than other specialists. CAS outcomes were not influenced by specialty but costless when performed by vascular surgeons (*P* < 0.0001).

**Conclusions:**

Structural factors affect CEA outcomes, but data on CAS were limited. These findings may inform reconfiguration of vascular services, reducing risks and costs associated with carotid interventions.

## Highlights

• The organization of hospitals may affect carotid interventional outcomes.
• Large vascular units with defined clinical pathways had improved surgical outcomes.
• Vascular surgeons had best outcomes after carotid interventions.
• These findings may reduce risks and costs associated with carotid interventions.

## Introduction

The structure of a health care system can impact on quality of patient care. Donabedian described how three factors involved in health care delivery interact to influence quality of outcome for a given patient.^1^ First, “structural factors” assess the systemic/organizational elements that reflect the setting within which care is delivered.^1^ Second, “process factors” assess the quality of care that a patient receives.^1^ For example, Hannan et al.^2^ demonstrated that use of shunts, patches, eversion, and protamine reduced complication rates after carotid endarterectomy (CEA). Third, “outcome measures” are useful for assessing the results of the intervention.^1^ Table I provides examples of these factors with their advantages and disadvantages.^3^

**Table I:** Examples, advantages, and disadvantages of the various components of the Donabedian model of health care structure

	Structural factors	Process factors	Outcome factors
Examples	• Number of hospital beds • Size of population served • Number of intensive care beds	• Patch closure of arteriotomy • Use of intraoperative heparin • Dual antiplatelet agents	• Mortality rate • Complication rate • Readmission rate
Advantages	• Readily measurable • Can have a large effect	• Easily assessed • Change can be made easily	• Adopted enthusiastically • Directly assess the result of the intervention
Disadvantages	• Re-organization required to institute changes • Isolating biggest contributor problematic (confounding)	• Evidence-based interventions may not be suitable for all patients • Large amount of data required to identify failures	• Large amount of data required to identify failures • ‘Hawthorne effect’—collecting outcomes improves them

A systematic review concluded that, in addition to measuring outcomes, there needs to be increased emphasis on assessing and optimizing structural and process factors to improve the quality of care delivered to vascular patients.^4^

### Optimizing Structural Factors Internationally

In the United States of America, the Institute of Medicine's Committee on Quality of Health Care's document *To Err Is Human: Building a Safer Health System* suggested implementing safeguards against harm due to structural factors.^5^ The National Center for Patient Safety was created to encourage a system-orientated approach to patient safety which encourages adverse event reporting as a tool for learning and improvement.^6^ *Crossing the Quality Chasm: A New Health System for the 21st Century*^7^ was published the following year and reinforced the need to prioritize patient safety in health care delivery.

The Vascular Society of Great Britain and Ireland recently published *The Provision of Services for Patients with Vascular Disease* document.^8^ This recommended centralization of services, so that a single “arterial hospital” would provide all arterial surgery, complex endovascular procedures, and receive acute referrals. This required: 24/7 on-call consultant vascular surgeons; a 24/7 surgical intensive care unit (SICU) and vascular anesthetist; 24/7 operating room; availability of dedicated vascular wards; and clearly defined clinical pathways.^8^ However, evidence for these proposed structural changes is currently limited.

### Aim

We aimed to examine the evidence for an association between structural factors (i.e. population size served by a vascular department; number of hospital beds; availability of dedicated vascular beds; clearly defined clinical pathways; SICU size; and the specialty of surgeon/interventionalist) and procedural outcomes (mortality; stroke; combined stroke/death; myocardial infarction (MI)/cardiac events; length of hospital stay (LOS); and cost) after CEA or carotid artery stenting (CAS).

## Materials and Methods

This systematic review is reported using the Preferred Reporting Items for Systematic Reviews and Meta-Analyses checklist.^9^ It was registered prospectively with international prospective register of systematic reviews (number: CRD42017081202). No changes were made to the analysis plan.

### Search Strategy

The databases of Medical Literature Analysis and Retrieval System Online, using the Ovid interface from 1946 onward and Excerpta Medica Database from 1974 onward, were searched (both last searched: July 31, 2019). Hand-searches of key journals and conference proceedings, as well as reference list searches of included titles were also conducted. Studies published before 2005 were excluded as these findings were not deemed relevant to current practice. This is similar to a recent systematic review's methodology.^10^ Abstracts, review articles, case reports, editorials, opinions, and commentaries were excluded as were studies examining the volume-outcome relationship as a systematic review have recently been published on this topic.^11^

**Fig. 1:**
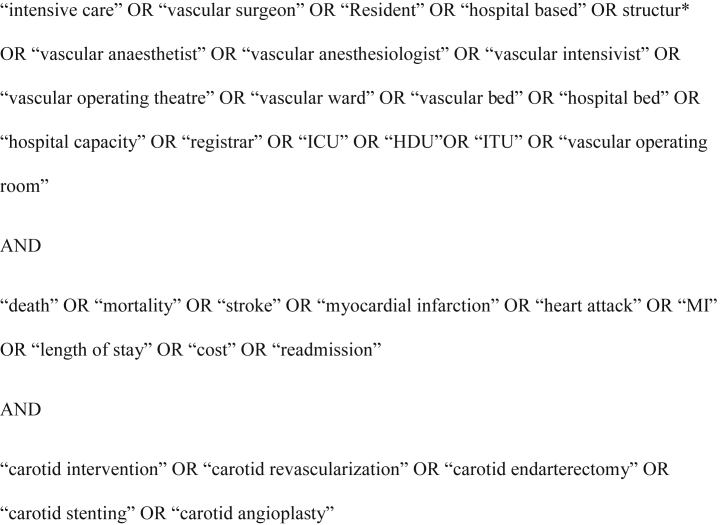
Search strategy for inclusion of studies in this systematic review.

The search strategy is detailed in Figure 1 in the following (after consultation with a librarian).

### Study Selection

**Fig. 2:**
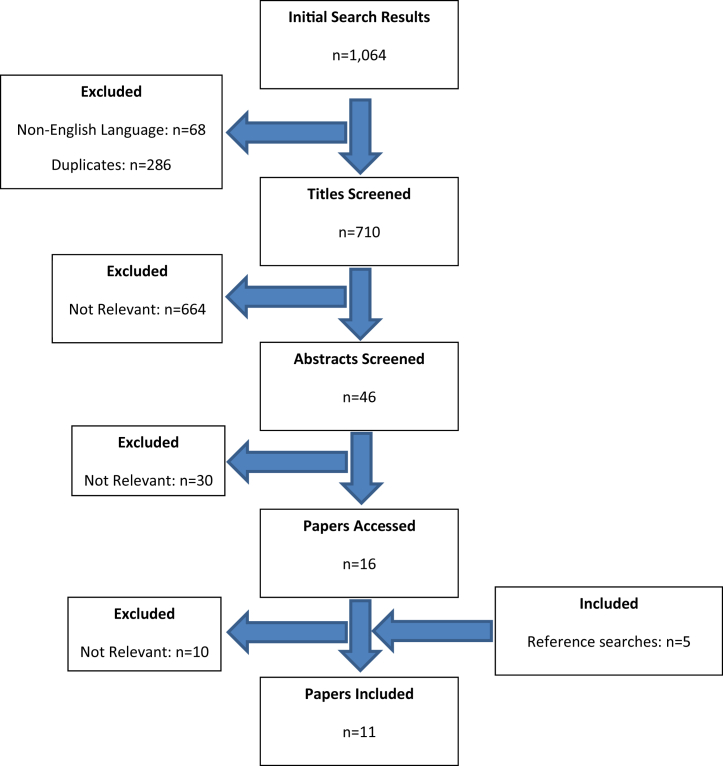
PRISMA diagram for the selection of studies in this systematic review.

The initial search yielded 1,064 titles. 68 titles were not in the English language, and 286 duplicates were also excluded. 710 titles were then screened by a single reviewer (KG) and ratified by a second reviewer (PC). Articles that were not relevant to the study's aim were excluded, yielding 46 remaining studies. The abstracts of these studies were screened independently by two reviewers (KG and DM) and both were blinded to the other's results. There was a 91.3% agreement between KG and DM (κ = 0.81; *P* < 0.00001). Any disagreements were adjudicated by a third reviewer (PC). This yielded 16 studies for full article review. Ten studies were deemed irrelevant and were subsequently excluded. Five studies were included after a search of the reference lists of the previously included articles. The whole process is summarized in Figure 2. These 11 articles were read independently by two reviewers (KG and PC). Data were extracted, and the quality of each study was assessed and documented on a preprepared *pro forma*.

### Data Collection

Data extracted included the following: authors, year of publication, years study was conducted, country of investigation, study design, type of intervention; number of centers, number of patients, symptom status, risk factor assessment, structural factor(s) assessed, and procedural outcomes.

Structural factors assessed included the following: population size served by the vascular department, number of hospital beds, availability of dedicated vascular beds, clearly defined clinical pathways, SICU size, and specialty of surgeon/interventionalist.

### Outcomes and Quality Assessment

Main outcomes included the following: 30-day rates of mortality, stroke, combined stroke/death, MI/cardiac events, LOS, and cost.

Quality of studies was assessed using the Newcastle-Ottawa scale for observational studies. A meta-analysis was not performed due to the heterogeneous nature of the included studies.

## Results

### Study Characteristics

Eleven studies from three countries, with a total of 95,100 patients, were included in this systematic review.^12^, ^13^, ^14^, ^15^, ^16^, ^17^, ^18^, ^19^, ^20^, ^21^, ^22^ All studies were retrospective, with no randomized controlled trials performed that were related to the study's aim (Table II).

**Table II:** Characteristics and quality of included studies. Quality was assessed by the Newcastle-Ottawa scale for observational studies

Authors	Year of publication	Years included in study	Country/countries	Study design	Intervention(s)	Number of centers	Sample size	Symptomatic/asymptomatic	Selection	Comparability	Exposure	Overall quality
Hussain et al.^18^	2018	2002–2015	Canada	Retrospective	CEA	1	16,544	Both	∗∗∗	∗∗	∗∗	∗∗∗∗∗∗∗
Chen et al.^14^	2016	2008–2009 & 2011–2012	Australia	Retrospective	CEA	1	117	Both	∗∗	∗	∗∗	∗∗∗∗∗
Enomoto et al.^15^	2014	2005–2010	USA	Retrospective	CEA	Not stated	34,493	Not stated	∗∗∗	∗∗	∗∗	∗∗∗∗∗∗∗
Abu Rahma et al.^12^	2014	Not stated	USA	Retrospective	CEA	1	1,000	Both	∗	∗	∗∗	∗∗∗∗
Paty et al.^19^	2014	2002–2012	USA	Retrospective	CEA	1	369	Symptomatic	∗∗	∗	∗∗	∗∗∗∗∗
Timaran et al.^21^	2013	2000–2008	USA & Canada	Retrospective	CAS & CEA	117	2,320	Both	∗∗∗	∗∗	∗∗	∗∗∗∗∗∗∗
Abu Rahma et al.^13^	2013	2010–2011	USA	Retrospective	CEA	1	953	Both	∗∗∗	∗	∗∗	∗∗∗∗∗∗
Gray et al.^16^	2011	2006–2009	USA	Retrospective	CAS	180	3,388	Asymptomatic	∗∗	∗	∗∗	∗∗∗∗∗
Hollenbeak et al.^17^	2010	1995–2002	USA	Retrospective	CEA	Not stated	17,627	Both	∗∗	∗	∗∗	∗∗∗∗∗
Steppacher et al.^20^	2009	2005–2006	USA	Retrospective	CAS	424	4,001	Both	∗∗∗	∗∗	∗∗	∗∗∗∗∗∗∗
Westvik et al.^22^	2006	1991–2002	USA	Retrospective	CEA	26	14,288	Both	∗∗∗	∗∗	∗∗	∗∗∗∗∗∗∗

CAS, Carotid Artery Stenting; CEA, Carotid Endarterectomy; UK, United Kingdom; USA, United States of America

Most studies analyzed CEA in both symptomatic and asymptomatic patients.

### Quality Assessment

The quality of included studies varied from four to seven stars on the Newcastle-Ottawa scale. There were five^15^^,^^18^^,^^20^, ^21^, ^22^ medium quality (7 stars) and six^12^, ^13^, ^14^^,^^16^^,^^17^^,^^19^ poor quality studies (≤6 stars) (Table II).

### Effect of Population Size, Bed Capacity, and Availability on CEA Outcomes

Vascular departments serving larger populations (>75,000 people) were associated with significantly reduced mortality (0.4% vs. 0.9%; *P* < 0.0001) and total stroke rate (1.0% vs. 1.8%; *P* = 0.001) after CEA than those serving smaller populations (Table III).^22^ There was no effect on MI/cardiac event rate (2.5% vs. 2.2%; *P* = 0.32).^22^

**Table III:** A summary of the effect of various structural factors on outcome after carotid endarterectomy

Structural factor	Study (Year)	Mortality rate	Total stroke rate	Minor stroke rate	Disabling/major stroke rate	MI/Cardiac event rate	Cost (USD)
Population size	Westvik et al. (2006)^22^	≤75K 0.9% >75K 0.4% (***P* < 0.0001**)	≤75K 1.8% >75K 1.0% (***P* = 0.001**)			≤75K 2.2% >75K 2.5% (*P* = 0.32)	
**Hospital bed capacity**	Westvik et al. (2006)^22^	≤131 beds 1.2% 132-279 beds 0.6% ≥280 beds 0.3% (***P* < 0.0001**) ≤131 beds **OR 2.78 (1.10–7.04) *P* = 0.03**	≤131 beds 2.2% 132–279 beds 1.4% ≥280 beds 1.1% (***P* = 0.0008**) ≤131 beds **OR 1.96 (1.32–2.90) *P* = 0.001**			≤131 beds 3.4% 132-279 beds 2.3% ≥280 beds 2.2% (***P* = 0.01**) ≤131 beds **OR 3.01 (1.45–6.24) *P* = 0.003** 132-279 beds **OR 2.15 (1.08–4.28) *P* = 0.03**	
Dedicated vascular beds	Westvik et al. (2006)^22^	Vascular beds 0.2% None 0.6% (***P* = 0.0008**)	Vascular beds 1.2% None 1.3% (*P* = 0.72)			Vascular beds 2.0% None 2.6% (***P* = 0.03**) **OR 1.35 (1.03–1.76) *P* = 0.03**	
Impact of pathway	Chen et al. (2016)^14^						616.73 saving per patient
	Paty et al. (2014)^19^	Pathway 0.0% No Pathway 0.0%	Pathway 3.1% No Pathway 7.9% (***P* = 0.03**)	Pathway 2.1% No Pathway 3.4%	Pathway 1.0% No Pathway 3.4%		
	Westvik et al. (2006)^22^	Pathway 0.5% No Pathway 0.5% (*P* = 0.90)	Pathway 1.0%, No Pathway 1.5% (***P* = 0.01**) **OR 1.39 (1.02–1.90) *P* = 0.04**			Pathway 2.0% No Pathway 2.7% (***P* = 0.004**) **OR 1.50 (1.19–1.89) *P* = 0.001**	
**ICU bed capacity**	Westvik et al. (2006)^22^	≤6 beds 0.8% ≥7 beds 0.3% (***P* < 0.0001**)	≤6 beds 1.7% ≥7 beds 1.1% (***P* = 0.001**)			≤6 beds 2.2% ≥7 beds 2.4% (*P* = 0.46)	
Dedicated ICU	Westvik et al. (2006)^22^	SICU 0.3% Mixed ICU 0.7% (***P* = 0.002**)	SICU 1.0% Mixed ICU 1.5% (***P* = 0.01**)			SICU 2.3% Mixed ICU 2.4% (*P* = 0.60)	
SICU admission	Westvik et al. (2006)^22^	Selective 0.3% Routine 0.6% (***P* = 0.01**)	Selective 1.2% Routine 1.3% (*P* = 0.63)			Selective 2.3% Routine 2.4% (*P* = 0.67)	
Dedicated intensivist	Westvik et al. (2006)^22^	Intensivist 0.4% No Intensivist 1.1% (***P* = 0.0002**)	Intensivist 1.2%, No Intensivist 1.7% (*P* = 0.12)			Intensivist 2.4% No Intensivist 2.2% (*P* = 0.65)	

Percentages are given to 1 decimal place, nonsignificant *P*-values, and costs (in US Dollars), and odds ratios (with 95% confidence intervals) are given to 2 decimal places; and significant *P*-values are highlighted and given to 1 significant figure.

ICU, intensive care unit; MI, myocardial infarction; OR, odds ratio; SICU, surgical intensive care unit; USD, US dollar.

Increased hospital bed capacity (≥280 beds) was independently associated with significantly reduced mortality (*P* < 0.0001), total stroke (*P* = 0.0008), and MI/cardiac event rate (*P* = 0.01) after CEA.^22^

Availability of dedicated vascular beds was associated with significantly reduced mortality (0.2% vs. 0.6%; *P* = 0.0008) and MI/cardiac event rate (2.0% vs. 2.6%; *P* = 0.03) after CEA but had no effect on total stroke rate.^22^

### Effect of Clinical Pathway on CEA Outcomes

Three studies examined the impact of an established clinical pathway on CEA outcomes (Table III).^14^^,^^19^^,^^22^ There was no difference in procedural mortality between groups that had a dedicated pathway and those that did not, but this was associated with a significantly reduced total stroke rate after CEA (1.0% vs. 1.5%; *P* = 0.01).^19^^,^^22^ Patients with an established pathway were also associated with significantly lower rates of MI/cardiac events after CEA than those without a pathway (2.0% vs. 2.7%; *P* = 0.004)^22^ while use of a clinical pathway was also associated with a cost saving per patient.^14^

### Effect of the SICU on CEA Outcomes

A larger (≥7 beds), dedicated SICU was associated with a significantly lower mortality (0.3% vs. 0.8%; *P* < 0.0001) and stroke rate (1.1% vs. 1.7%; *P* = 0.001) after CEA (Table III).^22^ SICU size had no effect on MI/cardiac event rate.^22^ Selective admission to ICU (based on predetermined criteria) halved mortality compared with routine admission (0.3% vs. 0.6%; *P* = 0.01) but had no effect on total stroke or MI/cardiac event rate after CEA.^22^

The presence of a dedicated intensivist was associated with an almost 3-fold reduced mortality rate after CEA (0.4% vs. 1.1%; *P* = 0.0002).^22^ Total nonfatal stroke and MI/cardiac event rates were similar between groups with and without a dedicated intensivist.^22^

### Effect of Specialty Performing CEA on Outcomes

Six studies examined the effect of various specialties on CEA outcomes (Table IV).^12^^,^^13^^,^^15^^,^^17^^,^^18^^,^^21^ 30-day mortality rates were similar for vascular and nonvascular surgeons performing CEA.^13^^,^^15^^,^^18^ While a significantly higher total stroke rate after CEA was associated with general surgeons, cardiothoracic surgeons, and neurosurgeons than vascular surgeons (2.4% vs. 1.6%; *P* = 0.01), 30-day minor stroke rates were similar between vascular surgeons, cardiothoracic surgeons, and general surgeons.^13^^,^^15^^,^^18^ A higher 30-day MI/cardiac event rate after CEA was associated with vascular surgeons than general surgeons performing the procedure (0.7% vs. 0.2%; *P* = 0.02).^15^^,^^21^

**Table IV:** A summary of the effect of various specialists performing the carotid intervention on outcome

Structural factor	Study (Year)	Mortality rate	Mortality/stroke rate	Total stroke rate	Minor stroke rate	Disabling/major stroke rate	MI/cardiac event rate	LOS (days)	Cost (USD)
VS performing CEA	Abu Rahma et al. (2014)^12^		VS 1.3% CTS 2.7% GS 4.1% (*P* = 0.11) non-VS 3.1% (***P* = 0.05**)						VS 1503.53 CTS/GS 2684.04
	Abu Rahma et al. (2013)^13^	VS 0.7% CTS 0.5% GS 0.0% (*P* = 1.00) non-VS 0.7% (*P* = 0.67)	VS 1.3% CTS 2.9% GS 4.1% (*P* = 0.13) non-VS 3.2% (*P* = 0.06)	VS 1.3% CTS 2.9% GS 4.1% (*P* = 0.13) non-VS 3.2% (*P* = 0.06)	VS 1.1% CTS 1.0% GS 0.0% (*P* = 0.80) non-VS 0.8% (*P* = 0.74)		VS 0.5% CTS 0.8% GS 0.0% (*P* = 0.83) non-VS 0.6% (*P* = 1.00)		
	Enomoto et al. (2014)^15^	VS 0.7% GS 1.1% (*P* = 0.11)		VS 1.6% GS 2.4% (***P* = 0.01**) OR 1.56 (1.13–2.17) ***P* = 0.008**			VS 0.7% GS 0.2% (***P* = 0.02**) OR 0.34 (0.12–0.90) ***P* = 0.03**	VS 2.7 GS 2.8 (*P* = 0.21)	
	Hollenbeak et al. (2010)^17^							VS 3.1 GS 3.5 (***P* = 0.0001)** CTS 3.2 NS 3.4 non-VS 3.1	VS 13,199.51 GS 12,659.87 (***P* = 0.0001**) CTS 12,448.30 NS 16,447.94 non-VS 13,165.04
	Hussain et al. (2017)^18^	VS 0.8% CTS 0.9% (*P* = 0.46) GS 1.2% (*P* = 0.18) NS 0.7% (*P* = 0.81) non-VS 0.9% OR 1.19 (0.8–1.76) *P* = 0.40	VS 2.9% CTS 4.4% **OR 1.54 (1.04–2.30) *P* = 0.03** GS 3.7% OR 1.33 (0.88–1.72) *P* = 0.22 NS 4.1% **OR 1.27 (1.00–1.61) *P* = 0.05** non-VS 4% (***P* = 0.008**)	VS 2.5% CTS 4.2% (***P* = 0.03**) GS 3.0% (*P* = 0.33) NS 3.8% (***P* = 0.009**) non-VS 3.6% **OR 1.38 (1.11–1.71) *P* = 0.004**					
	Timaran et al. (2013)^21^		VS 1.2% ± 0.4 non-VS 3.8% ± 0.9 **HR 0.32 (0.14–0.72) *P* = 0.006**				VS 2.6% non-VS 1.5%		
VS performing CAS	Gray et al. (2011)^16^	VS 1.3% (0.6–2.5) IC 0.6% (0.4–1.1) IN 1.5% (0.2–5.4) IR 0.5% (0.0–2.6) NS 0.0% (0.0–7.0)	VS 3.1% (1.9–4.7) IC 2.5% (1.9–3.2) IN 3.0% (0.8–7.6) IR 2.4 (0.8–5.5) NS 3.9% (0.5–13.5)	VS 2.7% (1.6–4.2) IC 1.9% (1.4–2.6) IN 1.5% (0.2–5.4) IR 1.9% (0.5–4.9) NS 3.9% (0.5–13.5)	VS 1.5% (0.7–2.7) IC 1.4% (1.0–2.0) IN 1.5% (0.2–5.4) IR 1.9% (0.5–4.9) NS 2.0% (0.0–10.4)	VS 1.2% (0.5–2.3) IC 0.6% (0.3–1.0) IN 0.0% (0.0–2.8) IR 0.0% (0.0–1.8) NS 2.0% (0.0–10.4)	VS 0.3% (0.0–1.1) IC 0.4% (0.2–0.7) IN 0.0% (0.0–2.8) IR 0.5% (0.0–2.6) NS 0.0% (0.0–7.0)		
	Steppacher et al. (2009)^20^^,^^a^	VS 0.9% IC 0.5% (*P* = 0.24) IR 0.5% (*P* = 0.18) non-VS 0.5% (*P* = 0.13)		VS 1.3% IC 1.1% (*P* = 0.59) IR 2.0% (*P* = 0.22) non-VS 1.5% (*P* = 0.73)				VS 2.8 ± 4.2 non-VS 3.0 ± 4.9 (*P* = 0.13)	VS 34,400.00 non-VS 40,600.00 (***P* < 0.0001**)
	Timaran et al. (2013)^21^		VS 3.8% ± 1.2 non-VS 4.5% ± 0.7 HR 1.12 (0.54–2.35) *P* = 0.76				VS 0.4% non-VS 0.9%		

Percentages are given to 1 decimal place; nonsignificant *P*-values, costs (in US dollars), and odds/hazard ratios (with 95% confidence intervals) are given to 2 decimal places, and significant *P*-values are highlighted and given to 1 significant figure.

CAS, carotid artery stenting; CEA, carotid endarterectomy; CTS, cardiothoracic surgeon; GS, general surgeon; HR, hazard ratio; IC, interventional cardiologist; IN, interventional neuroradiologist; IR, interventional radiologist; LOS, length of stay; MI, myocardial infarction; NS, neurosurgeon; OR, odds ratio; USD, US dollar; VS, vascular surgeon.

a This study assessed in-hospital outcomes whereas all other studies examined 30-day outcomes.

LOS after CEA was similar for patients treated by vascular surgeons or general surgeons in one study^15^ but significantly longer when treated by general surgeons in an older study (3.5 days vs. 3.1 days; *P* = 0.0001).^17^ CEAs performed by vascular surgeons were cheaper than for cardiothoracic/general surgeons in one study^12^ but significantly more expensive than general surgeons in an older study (*P* = 0.0001).^17^

### Effect of Specialty Performing CAS on Outcomes

Three studies reported similar procedural mortality, total stroke, combined stroke/death, minor stroke, disabling/major stroke, and MI/cardiac event rates after CAS performed by different interventionalists, neurosurgeons, and vascular surgeons (Table IV).^16^^,^^20^^,^^21^ LOS after CAS was comparable for patients treated by vascular surgeons (2.8 days) compared with nonvascular surgeons (3.0 days) while CAS performed by vascular surgeons was significantly cheaper (*P* < 0.0001).^20^

## Discussion

This is the first systematic review to assess the impact of structural factors on outcomes after carotid intervention comprehensively. The results suggest that improved CEA outcomes are achieved when large vascular departments serve larger populations, with established clinical pathways, large SICUs with dedicated intensivists, and vascular surgeons performing CEA. CAS outcomes are comparable between specialties but may be cheaper when performed by vascular surgeons. These important findings may help improve the configuration of vascular services and could reduce risks and costs associated with carotid interventions although data on CAS were limited.

### Effect of Population Size and Bed Capacity and Availability on CEA Outcomes

Our findings related to population size, hospital bed capacity, and dedicated vascular beds may be explained by the volume-outcome relationship. A strong positive correlation was demonstrated between hospital bed capacity and CEA volume,^22^ and population size is likely to determine the number of CEAs performed. As the volume-outcome relationship in CEA has been reported to reduce procedural mortality and stroke/death,^11^ this could explain our results. However, further analysis of this association is beyond the scope of this review.

### Effect of Pathway on CEA Outcomes

We found that an established clinical pathway was associated with significantly reduced total stroke and MI/cardiac event rate after CEA. This is not surprising as access to dedicated stroke unit personnel with neuro-ICU and immediate access to imaging^19^ may more effectively select and optimize patients for CEA, improving outcomes. A dedicated pathway to enable swift access to the cardiac angiography suite may also improve outcomes in patients who suffer an MI/cardiac event rate post-CEA.^22^ This may reduce costs per patient. Our findings support the establishment of clinical pathways to streamline patient care and improve CEA outcomes.^14^^,^^23^ However, this finding may also represent publication bias and therefore requires further investigation.

### Effect of the SICU on CEA Outcomes

Our finding that a large SICU with a dedicated intensivist was associated with decreased mortality and lower stroke rates after CEA may be due to increased nurse: patient ratio and invasive monitoring, allowing early detection of complications. While similar findings have been demonstrated in general surgery,^24^^,^^25^ our results may reflect outdated surgical practice as routine admission to SICU after CEA is not recommended currently.^22^^,^^26^, ^27^, ^28^, ^29^, ^30^

### Effect of Specialty Performing CEA on Outcomes

This systematic review reported a significantly lower stroke rate associated with patients treated by vascular surgeons than nonvascular surgeons. This is supported by older studies^2^^,^^31^^,^^32^ and might be due to vascular surgeons' techniques (e.g. preference for patch closure of arteriotomies).^12^ Our finding of a higher MI/cardiac event rate in cases operated by vascular surgeons than nonvascular surgeons contradicts the finding of another study^33^ and might possibly be due to case selection, as vascular surgeons’ patients had significantly higher rates of angina.^15^ When death/stroke/MI/cardiac events were combined, patients treated by vascular surgeons had significantly better outcomes than patients treated by nonvascular surgeons.^15^ Similar results have been reported in a recent systematic review and meta-analysis that was confounded by patient selection.^34^

Treatment by vascular surgeons was cheaper than nonvascular surgeons possibly because they used Doppler ultrasound as their investigation of choice preoperatively, compared with other specialties who may have preferred more expensive imaging modalities (such as computerized tomography/magnetic resonance angiography).

### Effect of Specialty Performing CAS on Outcomes

CAS outcomes were not influenced by the interventionalist's specialty in contrast to findings by Hopkins et al.^35^ who reported a two-fold increased 30-day risk of combined death/stroke/MI in patients undergoing CAS by vascular surgeons compared with interventional radiologists and interventional cardiologists. Our findings support current practice of CAS being performed by vascular surgeons, interventional radiologists, and interventional cardiologists (although data were limited) and are in keeping with the results from a recent systematic review and meta-analysis.^34^ The studies included in this systematic review may have failed to identify differences in CAS outcomes between specialties due to their limitations (inadequate power, underestimation of procedural strokes and overly strict credentialing).^16^^,^^20^^,^^21^ Further research is required to clarify this association.

Our finding that costs were significantly lower in patients undergoing CAS by vascular surgeons may again reflect their preference for cheaper preprocedural investigations.^20^

### Limitations

All included studies were retrospective and assessed short-term outcomes, with one study contributing to multiple associations.^22^ Administrative studies may have also had coding errors and studies were included from economically developed countries, limiting their generalizability. Centers contributing data to the included studies are also likely to be high-volume vascular departments, further limiting generalizability. However, most studies had large sample sizes and represent current practice. Data on CAS were also limited.

## Conclusion

Structural factors significantly affect procedural outcomes after CEA, although data on CAS were limited. These important findings highlight the need for an increased emphasis on optimizing structural factors to improve the configuration of vascular services and reduce the procedural risks and costs associated with carotid interventions.
